# Comparison of immune response to human rhinovirus C and respiratory syncytial virus in highly differentiated human airway epithelial cells

**DOI:** 10.1186/s12985-022-01805-2

**Published:** 2022-05-15

**Authors:** Xin-hui Yuan, Li-li Pang, Jing Yang, Yu Jin

**Affiliations:** 1 Medical School of Nanjing University, Nanjing, 210093 China; 2 Nanjing Children’s Hospital Affiliated to Nanjing Medical University, Nanjing, 210008 China; 3 The First Hospital of Lanzhou University, Lanzhou, 730000 China; 4 National Institute for Viral Disease Control and Prevention, Chinese Center for Disease Control and Prevention, Beijing, 102206 China

**Keywords:** Human rhinovirus C, Air–liquid interface, Human bronchial epithelial cell, Respiratory syncytial virus, Immune response, Cytokines

## Abstract

**Background:**

Human rhinovirus C (HRV-C) accounts for a large proportion of HRV-related illnesses, but the immune response to HRV-C infection has not been elucidated. Our objective was to assess the effect of HRV-C on cytokine secretion in human bronchial epithelial (HBE) cells grown at air–liquid interface (ALI) and compare it with that of respiratory syncytial virus (RSV).

**Methods:**

HBE cells were differentiated at ALI culture and the full-length cDNA clones of HRV-C651 and HRV-C15, clinical isolates of HRV-C79 and HRV-C101, and two RSV isolates were inoculated in the HBE cells. The effect of HRV-C on cytokine secretion was assessed and compared with that of RSV.

**Results:**

HRV-Cs infect and propagate in fully differentiated HBE cells and significantly increase the secretion of IFN-λ1, CCL5, IP10, IL-6, IL-8, and MCP-1. The virus loads positively correlated with the levels of the cytokines. HRV-C induced lower secretion of CCL5 (*P* = 0.048), IL-6 (*P* = 0.016), MCP-1 (*P* = 0.008), and IL-8 (*P* = 0.032), and similar secretion of IP10 (*P* = 0.214) and IFN-λ1 (*P* = 0.214) when compared with RSV.

**Conclusion:**

HBE ALI culture system supported HRV-C infection and propagation and HRV-C induced relatively weaker cytokine expression than RSV.

## Background

Human rhinovirus (HRV) infection causes many clinical diseases, including common cold, bronchiolitis, pneumonia, and asthma exacerbation, especially in children [1]. HRV is typically categorized into three species (HRV-A, HRV-B, and HRV-C) based on phylogenetic sequence analysis [2]. The recently discovered HRV-C is associated with a high proportion of HRV hospitalizations, and maybe more virulent, considering that HRV-C infections are overrepresented in children with pneumonia or acute wheezing and exacerbations of asthma [3, 4]. The role that HRV-C plays in these diseases suggests that it is important to understand host-specific or virus-specific factors that contribute to the pathogenesis.

The airway epithelial cells are the primary site of HRV infection. Exposure of airway epithelial cells to HRV leads to the release of a variety of proinflammatory cytokines and chemokines, which are related to pathogenic mechanisms and help to initiate an antiviral response. In contrast to HRV-A or -B, HRV-C cannot be propagated in immortalized cells. Due to difficulties in culturing this species, there is relatively little information on host cell responses to HRV-C infection. A handful of studies have described the successful amplification of HRV-C from clinical specimens in sinus mucosal organ cultures [5] and in differentiated sinus epithelial cells [6], as well as HRV-C generated from infectious clones in fully differentiated human airway epithelial (HAE) cells [7]. Although human bronchial epithelial cells (HBE) are the natural host for HRV infections, much of our understanding of how HRV replicates and induces immune responses is based on studies using non-airway cell lines (e.g., HeLa cells). HBE cells cultured in vitro at the air–liquid interface (ALI) form a pseudostratified epithelium that forms tight junctions and cilia and produces mucin, which provides a good representation of the airway epithelium in vivo [8].

Epidemiological studies suggest that RSV infection causes persistent wheezing and asthma [9], while HRV is more frequently involved in wheezing exacerbations in later childhood [10] and seems to be less harmful to bronchial structures compared with RSV. Therefore, we wondered whether these differences in disease characteristics may be accompanied by differences in the induction of cytokines or chemokines. To better understand HRV-C pathogenesis and the difference in immune responses between HRV-C and RSV infection in ALI HBE, we propagated two HRV-C viruses generated from infectious molecular clones of PC15 and Lz651 and two clinical specimens in fully differentiated human airway epithelial cells and analyzed the difference in cytokine secretion induced by HRV-C and RSV.

## Materials and methods

### Viruses and cells

Two plasmids, pCLZ651 and pC15, containing the full-length cDNA copies of HRV-C were a gift from Professor Zhao-jun Duan (China CDC, Beijing, China). Lz79 (GenBank: JF317014) and lz101 (GenBank: JF317017) were used as two clinical HRV-C samples. RSV1 (RSV-A1: ATCC-VR-1540) and RSV2 (RSV-B1: ATCC-VR-955) were used as RSV samples.

HeLa cells were cultured in DMEM supplemented with 10% FBS (Gibco, Grand Island, NY, USA) and 1% penicillin/streptomycin (Gibco) and were tested for mycoplasma (Mycoplasma Test Kit, ExCell Bio, Shanghai, China).

### Cultures of primary bronchial epithelial cells grown on ALI

Primary HBE cells were isolated from patients who underwent surgical lung resection for pulmonary diseases in Nanjing Children’s Hospital, as described previously [11]. HBE cells were plated onto type I and III collagen-coated six-well tissue culture plates and cultured in BEGM media (Lonza, Germany) supplemented with the required additives (Lonza). When the cells reached 80%–90% confluence, traditional monolayer two-dimensional (2D) HBE cells were dissociated using trypsin, and 3 × 10^5^ cells were seeded on type IV collagen-coated 12-well transwell inserts (Costar, ME, USA). The medium was renewed for both the apical and basolateral surfaces every other day. The medium was then changed to air–liquid interface (ALI) medium (BEGM + DMEM + additives) until the HBE cells reached full confluence. After 5 days, the HBE cells were exposed to air, and only the basolateral compartment was cultured in ALI medium. The ALI culture was continued for 4–6 weeks, during which time the cells differentiated into 3D pseudostratified HBE cells. Prior to the experiments, all cultures were maintained at 37 °C in a 5% CO_2_ incubator.

### In vitro HRV-C RNA transcription, transfection, and inoculation

The two plasmids, Lz651 and PC15, containing HRV-C viral genome were digested to linearize using Endonuclease Cal I (NEB), followed by HRV-C RNA transcription in vitro with a MEGAscript® T7 Transcription Kit (Ambion) in accordance with the manufacturer’s instructions. The RNA transcripts were treated with DNase I (Promega), purified with MEGAclear™ Transcription Clean-Up Kit (Ambion), and analyzed by formaldehyde denaturing agarose gel electrophoresis. RNA was transfected into HeLa cells with Lipofectamine 3000 (Invitrogen), and the dishes were incubated at 34 °C for 24 h. After freeze and thaw cycles, the virus was prepared. The cell lysates of PC15- and Lz651-transfected HeLa cells were added to the apical surface of the HBE 3D cells and incubated at 34 °C. Next, the HBE 3D cells were washed with PBS three times, and cells to assess cell-associated virus were collected in 500 µL Trizol (Invitrogen) at indicated time point.

### Immunostaining for HBE 3D cells and HRV-C

HBE 3D cells and those infected with 10^5^ RNA copies of HRV-C for 24 h were fixed in 4% paraformaldehyde (PFA) for 30 min at room temperature, followed by washing of both the apical and basolateral sides three times with PBS. Subsequently, the fixed cells were permeabilized with 0.2% Triton X for two hours and blocked with 5% bovine serum albumin for one hour. For the simultaneous detection of HRV-ssRNA, ciliated cells, secretion cells, and tight junctions, we applied anti-HRV-ssRNA mouse polyclonal antibody (mAb12, 10010200-200UG, 1:500, Thermo Fisher), mouse monoclonal anti-β tubulin antibody (T4026, 1:100, Sigma), MUC5AC monoclonal antibody (MAB11324,1:500, Abnova), and mouse monoclonal anti-ZO-1 antibody (33–9100, 1:500, Invitrogen), respectively. Light 594-labeled anti-mouse IgG (H + L) (35,511, 1:500, Thermo Fisher) was applied as the secondary antibody. To detect VP1 protein, rabbit polyclonal anti-HRV-C VP1 protein–antibody complexes (1:500, Invitrogen) bound to the cells were visualized using an Alexa Fluor 594 goat anti-rabbit IgG (1:500, Invitrogen). Nuclei were counterstained with DAPI. Finally, the filters with cells were excised from the insert and mounted under coverslips on glass slides using a mounting medium. Confocal images were taken with an UltraView VoX confocal microscope (PerkinElmer, Boston, MA, USA).

### Inoculation of other viruses on HBE 3D cells

HRV-C79 and HRV-C101 were quantified using qRT-PCR, and the RNA copies before inoculation were similar to those of pCLZ651 and pC15. RSV1 and RSV2 viral stock was propagated in HeLa cells; we infected HeLa cells with serially diluted RSV, followed by observation of cytopathic effects (CPE) to assess the 50% tissue culture infective doses (TCID50) per milliliter. TCID50 of RSV1and RSV2 were 10^−5^.

Virus suspensions of HRV-C79 and HRV-C101 (100 µL each) and RSV1 and RSV2 viral stocks (100 µL each) were applied on the apical surface of HBE 3D cells. At 4 h or 6 h post-incubation at 34 °C or 37 °C, the tissues were rinsed three times with PBS, and cultures were continued in the liquid–air interface in 500 μL of fresh culture medium.

### Quantitative real-time reverse transcription-PCR (qRT-PCR)

The RNAs of pCLZ651, pC15, HRV-C18, and HRV-C25 were quantified using a Quanti Tect Probe RT-PCR kit (QIAGEN) with the probe (FAM-TCC TCC GGC YCC TGA ATG-MGB) and the primers (HRV1A, 5′-AGC CTG CGT GGC TGC CTG-3′; HRV1A2, 5′-CCT GCG TGG CGG CCA RC-3′; HRV1B, 5′-CCC AAA GTA GTY GGT CCC RTC C-3′). These primers are complementary to the 5’ nontranslated regions of HRVsE1. For RSV1 and RSV2, the probe was FAM-CTGTGTATGTGGAGCCTTCGTGAAGCT; the forward primer was GGCAAATATGGAAACATACGTGAA; and the reverse primer was TCTTTTTCTAGGACATTGTAYTGAACAG. HRV-C RNA levels were normalized to human glyceraldehyde-3-phosphate dehydrogenase (GADPH) RNA levels in cell and tissue lysates with the probe (VIC-TGG TAT CGT GGA AGG A-MGB) and primers (forward, 5′-GCC AAA AGG GTC ATC ATC TC-3′; reverse, 5′-GGG GCC ATC CAC AGT CTT CT-3′).

### Cytokine and/or chemokine production

Basal medium was assayed for IFN-λ1, CCL5, IP10, IL-6, IL-8, and MCP-1 using solitary ELISA kits (eBioscience, USA) following the manufacturer’s instructions. Determination of each cytokine level was repeated three times.

### Statistical analysis

Statistical analysis was performed with SPSS, version 22.0. Data were presented as mean ± standard deviation. Mann–Whitney test for comparisons between two viral groups was used to determine statistical significance (*P* < 0.05).

## Results

### Immunofluorescence analysis for HBE 3D cells

**Fig. 1:**
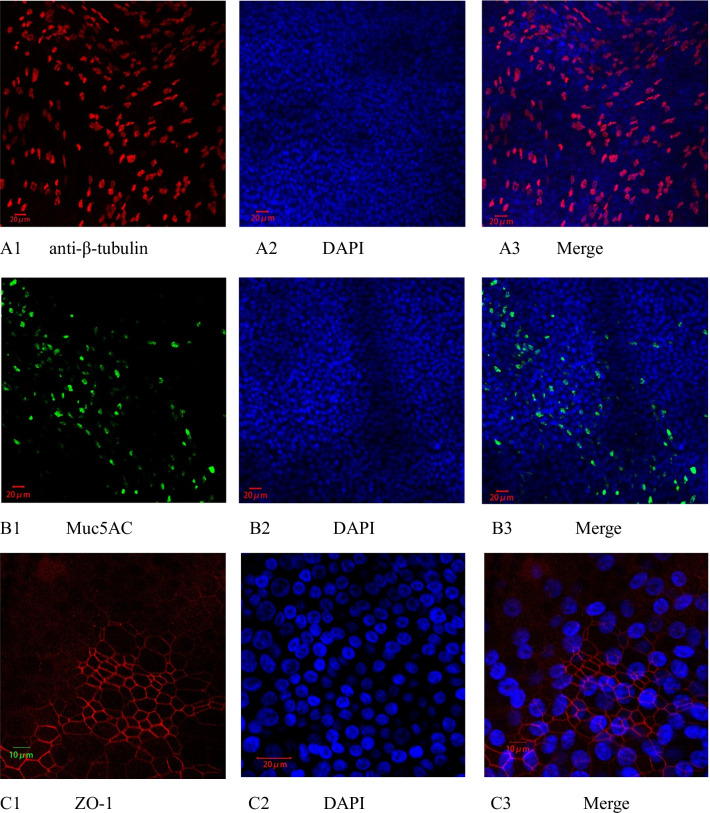
Immunofluorescence of differentiated HBE cells. The differentiated HBE cells were fixed, followed by incubation with mouse monoclonal anti-β tubulin antibody (red, **A**), mouse Muc5AC monoclonal antibody (green, **B**), or monoclonal anti-ZO-1 antibody (red, **C**). Confocal images were taken with a magnification of 200× for **A** and **B**, and 360× for **C**. Nuclei were stained with DAPI. Scale bars, 20 μm

β-tubulin is an important component of the cytoskeleton and a marker protein of cilia, so it can be used to distinguish between ciliated cells and nonciliated cells. The tight junction protein zonula occludens-1 (ZO-1) is located at the apex of epithelial cells; it is a major component of tight junctions and plays an important role in maintaining pseudostratified cell layer integrity and barrier function. As shown by immunofluorescence staining with anti-β-tubulin IV (Fig. 1A), Muc5AC (Fig. 1B), and anti-ZO1 (Fig. 1C) antibodies, the ciliated cells were well differentiated, and the number of secretory cells was large. In addition, the ZO-1 protein formation was good, indicating that the tight junctions of the differentiated epithelial were well-formed.

### HRV-C infection in HBE-ALI

**Fig. 2:**
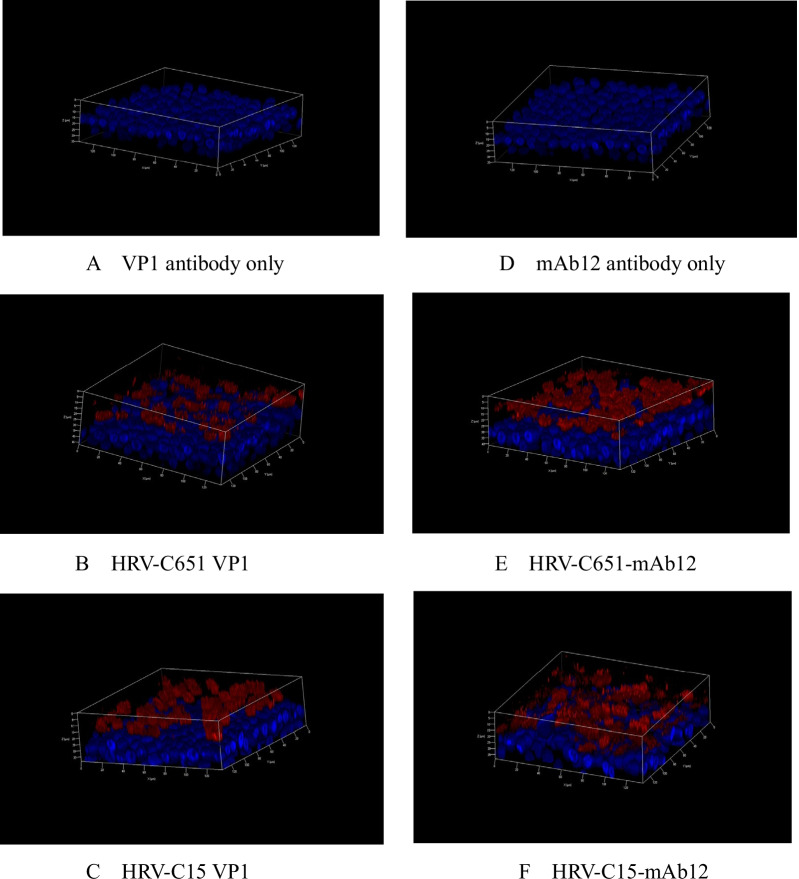
Immunofluorescence of differentiated HBE cells infected with HRV-C651 or HRV-C15. Immunofluorescence with the rabbit polyclonal anti-HRV-C VP1 protein-antibody only (**A**) and mAbJ2 antibody (**D**). At 24 h post-inoculation with HRV-C651 or HRV-C15, the HBE cells were fixed, followed by incubation with VP1 protein-antibody (**B** and **C**) and mAb12 antibody detecting double-strand RNA (**E** and **F**). The red region indicates VP1 protein (**B** and **C**) or the double-stranded RNA of HRV-C (**E** and **F**). Confocal images were taken with a magnification of 200×. Nuclei were stained with DAPI

The full-length cDNA clones of PC15and LZ651 were transcribed in vitro and transfected into HeLa cells. To determine whether the viral particles infected HeLa cells and propagated after transfection of HeLa cells with HRV-C651 or HRV-C15 RNA, we used HRV-C VP1 antibody and a murine monoclonal antibody J2—widely used antibodies for the detection of rhinovirus double-stranded RNA. HBE 3D cells were incubated with the lysate of viral RNA or mock-transfected cells. Rhinovirus VP1 capsid protein and double-stranded RNA expression levels were monitored using an immunofluorescence assay (Fig. 2). The localization of infected cells confirmed that viral replication was restricted to the apical surface of the HBE-ALI.

### HRV-C and RSV replicate in HBE-ALI

**Fig. 3:**
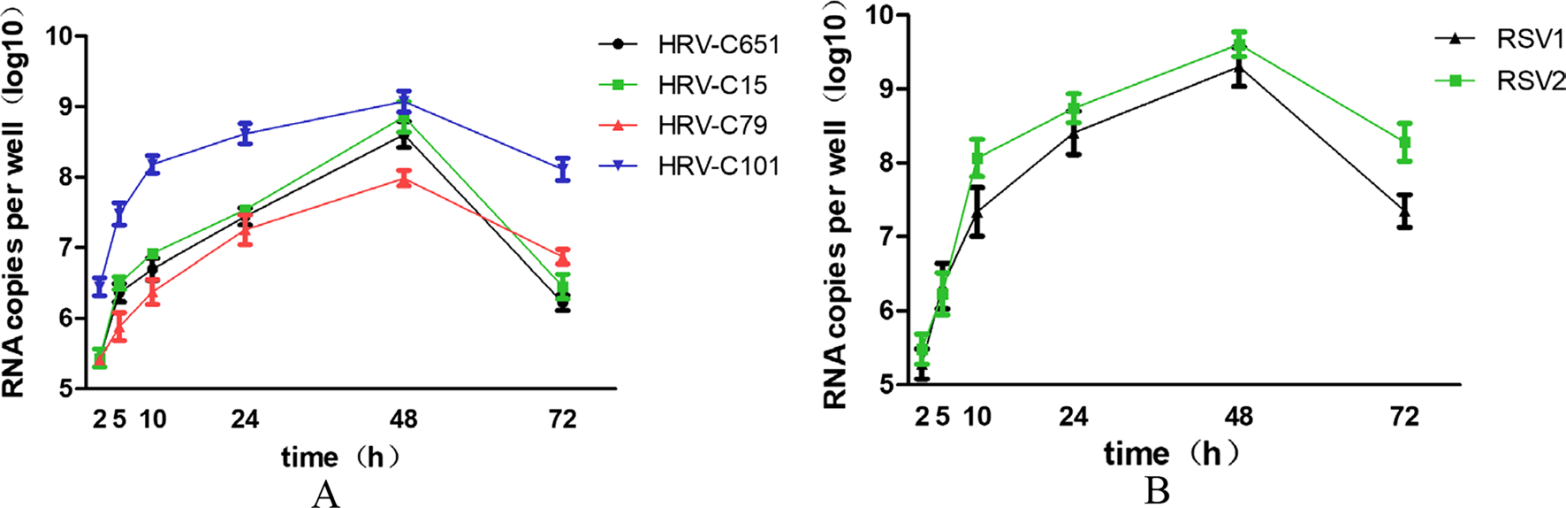
Four HRV-C strains and two RSV strains grew successfully in ALI HBE cells (**A** and **B**). Virus RNA load from infected tissue supernatants was measured by real-time RT-PCR collected at the apical surface of ALI HBE cells infected with HRV-C strains (HRV-C 651, HRV-C15, HRV-C 79, or HRV-C 101) and two RSV strains (RSV1 or RSV2). Error bars represent the standard deviation calculated from biological replicates (n = 3)

To verify whether HRV-C and RSV replicate in differentiated HBE cells, lysates of HeLa cells transfected with HRV-C651 or HRV-C15 RNA and RSV virus stock were incubated at the apical side of the HBE-ALI cells. Then virus productions were quantified by real-time RT-PCR from the apical side and basal sides at different times post-infection, and virus RNA at the apical surface was detected significantly rise but not at the basal surface. We found that HRV-Cs (including HRV-C651, HRV-C15, HRV-C79, and HRV-C101) propagated considerably at 5 h post-infection (PI), with peak RNA levels achieved 48 h after infection (Fig. 3A). Of note, 48 h PI, the RNA level of HRV-C651, HRV-C15, HRV-C79, and HRV-C101 increased significantly from 4.23 to 8.35, 4.64 to 8.49, 5 to 7.95, and 5.45 to 9.08 log10 copies/well, respectively. RSV propagated significantly at 10 h PI, with a peak level achieved at 48 h PI (Fig. 3B). The RNA level of RSV1and RSV2 virus increased from 5.2 to 9.3 and from 5 to 9.5 log10 copies/well, respectively (178-fold and 190-fold, respectively).

### Effects of HRV-C on induction of cytokines and comparison with RSV

**Fig. 4:**
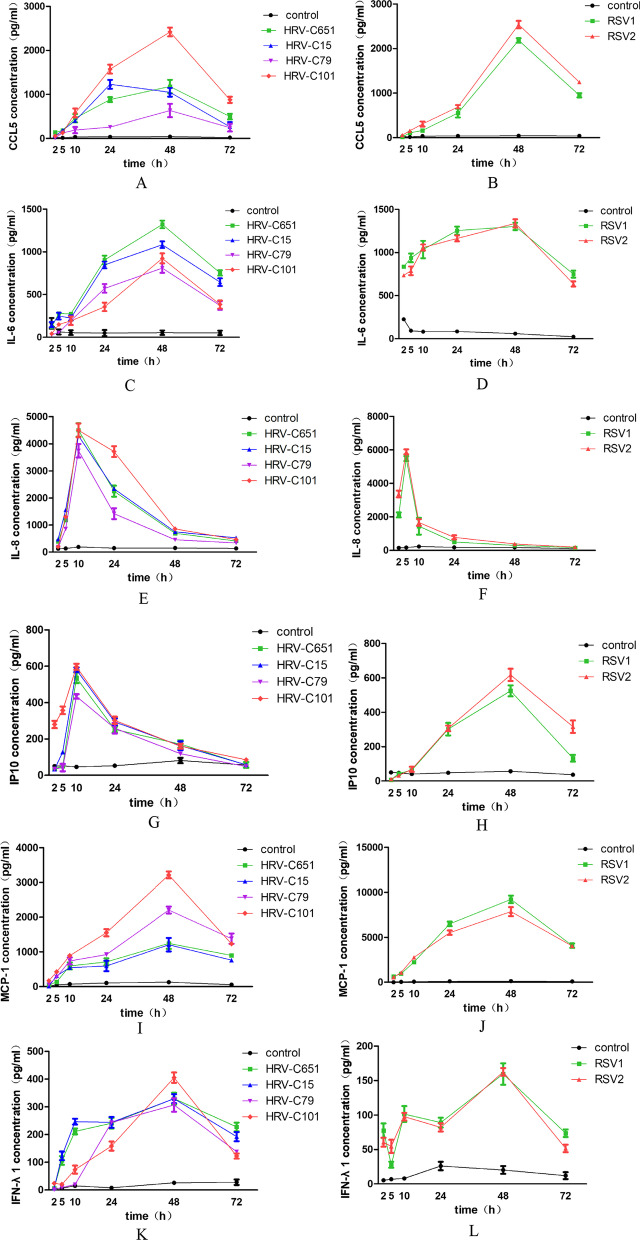
Time course of cytokine production induced by HRV-Cs and RSVs. Levels of cytokines in basal medium of HBE cultures were collected at each hour after HRV-Cs or RSVs inoculation in ALI HBE cells

Next, we examined the effects of HRV-C on the secretion of cytokines and compared them with those of RSV. We detected interferon (IFN-α, IFN-γ, IFN-λ), chemokines (CCL5, IP10), interleukin (IL-1β, IL-4, IL-5, IL-6, IL-8, IL12, L33), thymic stromal lymphopoietin (TSLP), eotaxin, eosinophilia cationic protein (ECP), monocyte chemoattractant protein-1, (MCP-1), and tumor necrosis factor α (TNF-α). In general, HRV-C infection induced the secretion of IFN-λ1, CCL5, IP10, IL-6, IL-8, and MCP-1. The RNA copies number of HRV-C and RSV at every time point PI positively correlated with the concentrations of IL-8, IL-6, MCP-1, IFN-λ1, and CCL5. The protein levels of CCL5, IL-6, MCP-1, and IFN-λ1 induced by HRV-C reached the maximum at 48 h PI, while IL-8 and IP10 reached maximum at 5- and 10-h PI, respectively. The production of CCL5, IL-6, MCP-1, IP10, and IFN-λ1 induced by RSV reached the maximum at 48 h PI, while IL-8 peaked at 10 h PI (Fig. 4A–L).

**Fig. 5:**
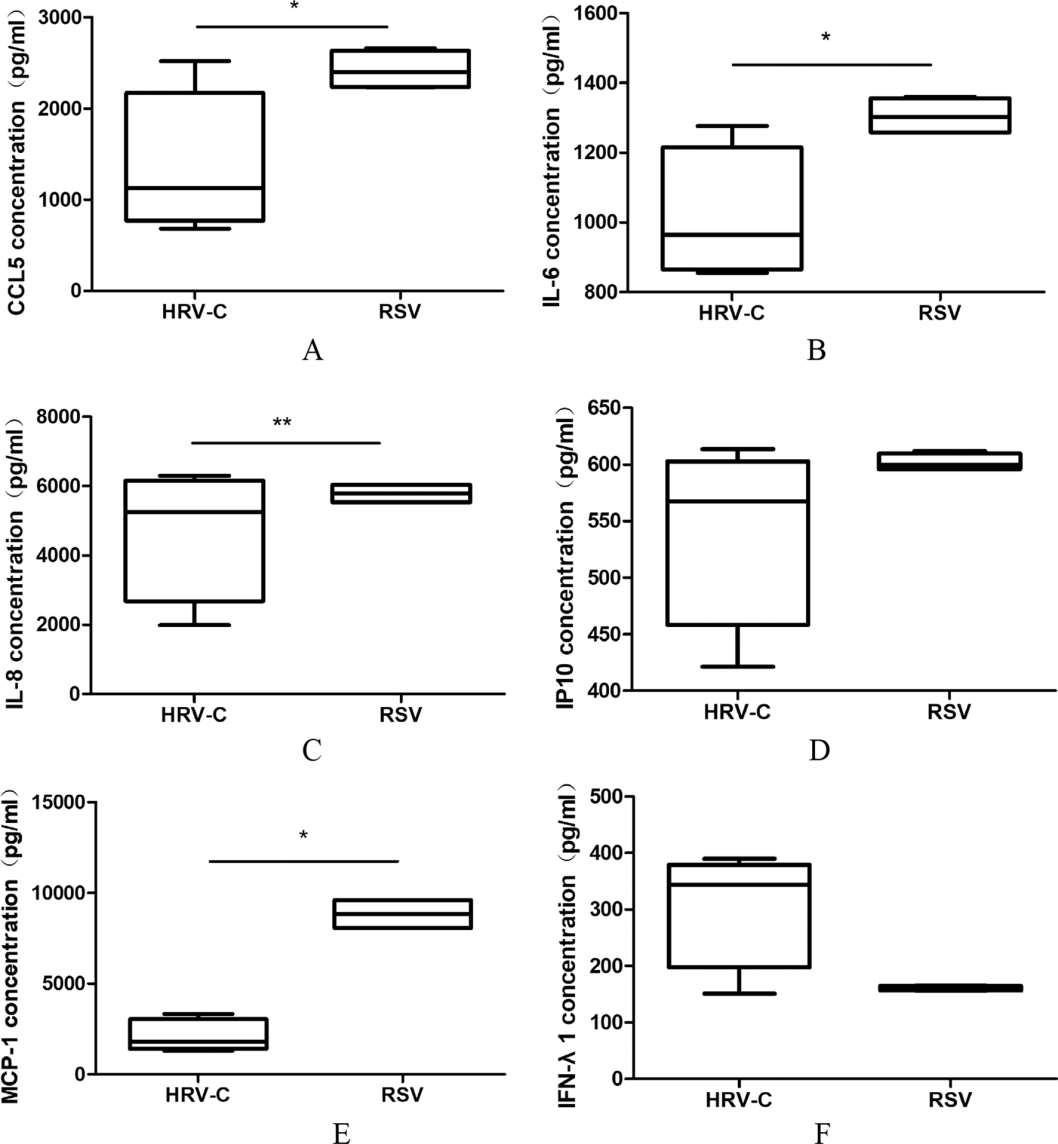
Comparison cytokine levels (mean ± SEM) between HRV-Cs and RSVs within 48-h PI in ALI HBE cells. Mann–Whitney test for comparisons between two viral groups was used to determine statistical significance. *P < 0.05, **P < 0.01

Furthermore, we evaluated the average values of cytokine secretion induced by HRV-C within 48 h PI. Compared with RSV, HRV-C induced lower secretion of CCL5 (*P* = 0.048), IL-6 (*P* = 0.016), IL-8 (*P* = 0.032) and MCP-1 (*P* = 0.008), and similar secretions of IP10 (*P* = 0.214), and IFN-λ1 (*P* = 0.214) (Fig. 5A–F).

## Discussion

Most in vitro studies on the response of human immune cells to HRV infection have used H1HeLa cells or submerged cultures of bronchial epithelial cell lines. In this study, we used ALI-cultured HBE cells, which can form tight junctions, produce mucin, and differentiate to form cilia, providing a good representation of the airway epithelium in vivo. CDHR3, a receptor for HRV-C, is only expressed on ciliated cells; therefore, undifferentiated airway epithelial cells may not be useful in studying responses to HRV-C unless the cells are genetically modified [12].

In this study, we transcribed the whole genome sequence of the C-type rhinovirus strains LZ651 and PC15 in vitro into viral mRNA and harvested two HRV-C infectious clones: HRV-C15 and HRV-C651. Then, HRV-C15 and HRV-C651, as well as clinical species HRV-C79 and HRV-C101 were inoculated into HBE cells under ALI conditions. We demonstrated RNA replication and virus protein expression after transfection with full-length HRVC-651 and HRVC15 RNA; the RNA level peaked at 48 h PI, increasing 183-fold and 197-fold for HRVC-651 and HRVC15, respectively. Likewise, HRV-C79 and HRV-C101 RNA levels increased 159-fold and 166-fold, respectively. Nakagome propagated three HRV-C full-length clones in differentiated sinus epithelial cells and showed that the RNA maximum was reached at 24 h PI, while our previous studies showed that HRV-C 651 RNA level peaked at 48 h PI [11, 13]. Tapparel et al. propagated eight HRV-C clinical strains in ALI HAE and displayed RNA load maximum between 24 and 72 h PI [5]. The different growing times of HRV-Cs in HBE differentiated cells represented type-dependent. Airway epithelial cells are the primary site of rhinovirus replication and are responsible for initiating the host immune response to infection. Rather than cytotoxic effects, experimental or naturally occurring exposure of airway epithelial cells to HRV normally induces a virus-specific cytopathic effect, which is associated with an inflammatory reaction [14]. Although we tested a variety of cytokines and chemokines associated with HRV-C infection according to previous data, only a few cytokines’ levels significantly increased [15]. A similar result was observed in Nakagome et al.’s study [13]. Souza demonstrated that undifferentiated cells showed increased expression of a variety of inflammatory cytokines in response to HRV-A16 infection, but well-differentiated cells did not respond [16]. This phenomenon indicates that well-differentiated cells are much more resistant to viral infection and its functional consequences than poorly differentiated cells from the same source [16]. We found that HRV-C infection in HBE ALI cells significantly stimulated the secretion of CCL5, IL-6, IL-8, IP-10, MCP-1, and IFN-λ1, and these protein levels positively correlated with viral proliferation, suggesting that these cytokines are involved in the immune response against HRV-C infection. This presumably represents a defensive response on the part of the epithelium, for all these cytokines are capable found in HRV-C infection in HBE ALI cells. A difference between the HRV-C strains both the cytokines induced and the amounts may mainly attribute to the different resources of HRV-C strains.

HRV and RSV are two leading etiologies of acute respiratory diseases, and the epithelium of the airways is their main target [15]. Despite its lower cytotoxicity compared with RSV, HRV induces the activation of the airway cells with subsequent release of proinflammatory cytokines. Compared with RSV infection, HRV-C induced relatively low cytokine secretion (except IFN-λ1 and IP-10) in response to HBE ALI cells during 48 h in this study. This is in accordance with previous clinical studies [17, 18]. Unlike RSV, HRV does not determine airway epithelial cell a clear cytopathic effect but compromises the epithelial barrier function by dissociating zonula occludens-1 of the cells from the tight junction complex during viral replication [19]. A weaker response of HBE to HRV-C may be attributed to the fact that HRV-C causes less cell damage and has lower cytotoxicity. Because of the production of IL-6 and IL-8, RANTES can lead to airway damage, neutrophils-mediated epithelial damage, and bronchial hyper-responsiveness [15].

In the present study, compared with RSV, IL-8 was rapidly induced after HRV-C inoculation, which occurred earlier than the time that it takes to amplify the maximum amount of HRV-C RNA and other cytokines. In contrast, the production of IL-6 peaked at 48 h after HRV-C infection. The levels of IL-6 and IL-8 were lower than those induced by RSV. The maximum levels of CCL5 after HRV-C infection were achieved at 24- or 48-h PI, and the protein levels were lower than those of RSV. Yamaya et al. demonstrated that HRV-14 (HRV-A) infection released IL-6 and IL-8, and the levels were higher in the supernatants of the cells obtained from subjects with bronchial asthma than in those from the non-asthmatic group [20]. Chun et al. [21] infected HRV serotype 7 in A549 human airway epithelial cells and found that HRV caused greater IL-8 and CCL5 release than RSV. This may be due to the different culture systems and virus types. IL-8 acts as a key mediator in neutrophil activation and is believed to contribute to airway obstruction following respiratory viral infection [22]. An increase in neutrophil counts has been observed in the lower airways of infants with recurrent wheezing and IL-8 production has been found in acute exacerbations of asthma induced by HRV [23]. RANTES is involved in the chemoattraction of eosinophils, monocytes, and T lymphocytes, and it is present in the respiratory secretions of patients with asthma [24]. IP-10 is a chemokine secreted by bronchial epithelial cells, monocytes, lymphocytes, and neutrophils in response to IFN and TNF; its levels are high during HRV infection. HRV-A infection in human airway epithelial cells increases IP-10 protein in vitro and in vivo [25] and may alter the host cytokine environment by leading to a persistent cytokine elevation, such as IP-10 gene expression [26]. As recently described in a paper by Shariff et al. [27], recurrent HRV infections are a strong stimulus for airway remodeling through an increase in smooth muscle cell mass recruitment next to the epithelial cells, which is mediated by CCL5, CXCL8, and IP-10 secreted during HRV infection. In the present study, IP-10 increased significantly after HRV-C infection in HBE ALI cells at 10 h PI, and the levels were similar to those after RSV infection. Both HRV-C and RSV induced high levels of IL-8, IL6, CCL5, and IP-10. Moreover, early clinical studies showed that HRV-C was associated with wheezing episodes [28, 29]. Our findings together with previous data might clarify the higher incidence of subsequent wheezing and asthma development after HRV-C bronchiolitis.

Airway epithelial cells express type I and type III interferons (IFNs), such as IFN-λ1, λ2, and λ3, in response to replication-efficient HRV infection. We found higher levels of IFN-λ1 after HRV-C infection than after RSV infection. Recent studies have implicated that RSV pathogenesis and immune responses are determined by type I IFN, and RSV is a poor inducer of IFN [30, 31]. In addition, a previous study that compared the HAE immune response to RSV and influenza virus infection showed that type III IFNs (IL-28 and IL-29) induced by RSV were absent [32]. Nevertheless, Miller et al. demonstrated that asthma exacerbation associated with HRV infection was mainly mediated by an increase in type III IFN response [33]. These data might be helpful to understand the dissimilarity of antiviral responses to HRV-C infection and RSV infection.

MCP-1 is a crucial mediator of monocyte chemotaxis and T-lymphocyte differentiation, with a key role in the pathogenesis of several conditions. In patients with asthma, an increased expression of MCP-1 has been reported with the activation of a dysregulated Th2 response [34]. Inhibition of MCP-1 expression significantly reduced airway reactivity in an experimental model of asthma [35]. A study by Giuffrida has shown that MCP-1 was significantly increased in asthmatic patients when compared with non-asthmatic patients, and only RSV induced a significant increase in MCP-1 expression, when compared with parainfluenza virus and adenovirus [34]. Our study found that MCP-1 protein was significantly increased in both HRV-C and RSV infections, but HRV-C infection induced lower level than RSV. These results support the clinical data that rhinovirus is responsible for 50% of asthma exacerbation, and RSV has been associated with recurrent wheezing and asthma development [36].

The limitation of this study is that we compared data of HRV-C infectious clones or strains from clinical samples with RSV experimental strains. However, we found that the immune response to virus infections between infectious clones and clinical samples was similar. In addition, We only used a blank control, not a UV-inactivated virus to evaluate the impact of viral replication death variants. Viral stocks were applied on the apical surface of HBE 3D cells and incubated for four hours, and the tissues were rinsed three times with PBS, which could reduce the effect of viral replication death variants.

## Conclusion

Our study demonstrated that the HBE ALI culture system supported HRV-C infection and propagation. When compared with RSV, HRV-C induced relatively weaker cytokine expression in fully differentiated HBE cells. The analysis of the difference in immune response between the two viruses may be helpful for the development of therapies and preventive strategies.

## Data Availability

The data sets used and/or analyzed during the current study are available from the corresponding author on reasonable request.

## Abbreviations

HRV: Human rhinovirus
HRV-C: Human rhinovirus C
HBE: Human bronchial epithelial
ALI: Air–liquid interface
RSV: Respiratory syncytial virus
HAE: Human airway epithelial
2D: Two-dimensional
RT-PCR: Reverse transcription-PCR
PI: Post infection
IFN: Interferon
IL: Interleukin
TSLP: Thymic stromal lymphopoietin
ECP: Eosinophilia cationic protein
MCP-1: Monocyte chemoattractant protein-1
TNF-α: Tumor necrosis factor α
ZO-1: Zonula occludens-1

## References

[1] Jacobs SE, Lamson DM, St. George K, Walsh TJ (2013). Human Rhinoviruses. Clin Microbiol Rev.

[2] Palmenberg AC, Spiro D, Kuzmickas R, Wang S, Djikeng A, Rathe JA, Fraser-Liggett CM, Liggett SB (2009). Sequencing and analyses of all known human rhinovirus genomes reveal structure and evolution. Science.

[3] Smuts HE, Workman LJ, Zar HJ: Human rhinovirus infection in young African children with acute wheezing. 10.1186/1471-2334-11-65 PMC3065410 21401965

[4] Mak RK, Lai YT, Lam WY, Wong GWK, Chan PKS, Leung TF: Clinical spectrum of human rhinovirus infections in hospitalized Hong Kong children. 10.1097/INF.0b013e31821b8c71 21494174

[5] Tapparel C, Sobo K, Constant S, Huang S, Van Belle S, Kaiser L (2013). Growth and characterization of different human rhinovirus C types in three-dimensional human airway epithelia reconstituted in vitro. Virology.

[6] Ashraf S, Brockman-Schneider R, Bochkov YA, Pasic TR, Gern JE (2013). Biological characteristics and propagation of human rhinovirus-C in differentiated sinus epithelial cells. Virology.

[7] Hao W, Bernard K, Patel N, Ulbrandt N, Feng H, Svabek C, Wilson S, Stracener C, Wang K, Suzich J (2012). Infection and propagation of human rhinovirus C in human airway epithelial cells. J Virol.

[8] Dvorak A, Tilley AE, Shaykhiev R, Wang R, Crystal RG (2011). Do airway epithelium air-liquid cultures represent the in vivo airway epithelium transcriptome?. Am J Respir Cell Mol Biol.

[9] Blanken MO, Rovers MM, Molenaar JM, Winkler-Seinstra PL, Meijer A, Kimpen JL, Bont L (2013). Respiratory syncytial virus and recurrent wheeze in healthy preterm infants. N Engl J Med.

[10] Vandini S, Biagi C, Fischer M, Lanari M (2019). Impact of rhinovirus infections in children. Viruses.

[11] Pang LL, Yuan XH, Shao CS, Li MZ, Wang Y, Wang HM, Xie GC, Xie ZP, Yuan Y, Zhou DM (2017). The suppression of innate immune response by human rhinovirus C. Biochem Biophys Res Commun.

[12] Griggs TF, Bochkov YA, Basnet S, Pasic TR, Brockman-Schneider RA, Palmenberg AC, Gern JE (2017). Rhinovirus C targets ciliated airway epithelial cells. Respir Res.

[13] Nakagome K, Bochkov YA, Ashraf S, Brockman-Schneider RA, Evans MD, Pasic TR, Gern JE (2014). Effects of rhinovirus species on viral replication and cytokine production. J Allergy Clin Immunol.

[14] Rossi GA, Colin AA (2015). Infantile respiratory syncytial virus and human rhinovirus infections: respective role in inception and persistence of wheezing. Eur Respir J.

[15] Vandini S, Calamelli E, Faldella G, Lanari M (2017). Immune and inflammatory response in bronchiolitis due to respiratory syncytial virus and rhinovirus infections in infants. Paediatr Respir Rev.

[16] Lopez-Souza N, Dolganov G, Dubin R, Sachs LA, Sassina L, Sporer H, Yagi S, Schnurr D, Boushey HA, Widdicombe JH (2004). Resistance of differentiated human airway epithelium to infection by rhinovirus. Am J Physiol Lung Cell Mol Physiol.

[17] Roh DE, Park S-H, Choi HJ, Kim YH (2017). Comparison of cytokine expression profiles in infants with a rhinovirus induced lower respiratory tract infection with or without wheezing: a comparison with respiratory syncytial virus. Korean J Pediatr.

[18] Diaz PV, Valdivia G, Gaggero AA, Bono MR, Zepeda G, Rivas M, Uasapud P, Pinto RA, Boza ML, Guerrero J (2015). Proinflammatory cytokines in nasopharyngeal aspirate from hospitalized children with respiratory syncytial virus infection with or without rhinovirus bronchiolitis, and use of the cytokines as predictors of illness severity. Medicine (Baltimore).

[19] Unger BL, Ganesan S, Comstock AT, Faris AN, Hershenson MB, Sajjan US (2014). Nod-like receptor X-1 is required for rhinovirus-induced barrier dysfunction in airway epithelial cells. J Virol.

[20] Yamaya M, Nomura K, Arakawa K, Sugawara M, Deng X, Lusamba Kalonji N, Nishimura H, Yamada M, Nagatomi R, Kawase T (2020). Clarithromycin decreases rhinovirus replication and cytokine production in nasal epithelial cells from subjects with bronchial asthma: effects on IL-6, IL-8 and IL-33. Arch Pharm Res.

[21] Chun YH, Park JY, Lee H, Kim HS, Won S, Joe HJ, Chung WJ, Yoon JS, Kim HH, Kim JT, Lee JS (2013). Rhinovirus-infected epithelial cells produce more IL-8 and RANTES compared with other respiratory viruses. Allergy Asthma Immunol Res.

[22] Castillo JR, Peters SP, Busse WW (2017). Asthma exacerbations: pathogenesis, prevention, and treatment. J Allergy Clin Immunol Pract.

[23] Jartti T, Bonnelykke K, Elenius V, Feleszko W (2020). Role of viruses in asthma. Semin Immunopathol.

[24] Conti P, DiGioacchino M (2001). MCP-1 and RANTES are mediators of acute and chronic inflammation. Allergy Asthma Proc.

[25] Spurrell JC, Wiehler S, Zaheer RS, Sanders SP, Proud D (2005). Human airway epithelial cells produce IP-10 (CXCL10) in vitro and in vivo upon rhinovirus infection. Am J Physiol Lung Cell Mol Physiol.

[26] Wood LG, Powell H, Grissell TV, Davies B, Shafren DR, Whitehead BF, Hensley MJ, Gibson PG (2011). Persistence of rhinovirus RNA and IP-10 gene expression after acute asthma. Respirology.

[27] Shariff S, Shelfoon C, Holden NS, Traves SL, Wiehler S, Kooi C, Proud D, Leigh R (2017). Human rhinovirus infection of epithelial cells modulates airway smooth muscle migration. Am J Respir Cell Mol Biol.

[28] Müller L, Mack I, Tapparel C, Kaiser L, Alves MP, Kieninger E, Frey U, Regamey N, Latzin P (2015). Human rhinovirus types and association with respiratory symptoms during the first year of life. Pediatr Infect Dis J.

[29] Tran DN, Trinh QD, Pham NT, Pham TM, Ha MT, Nguyen TQ, Okitsu S, Shimizu H, Hayakawa S, Mizuguchi M, Ushijima H (2016). Human rhinovirus infections in hospitalized children: clinical, epidemiological and virological features. Epidemiol Infect.

[30] Marr N, Wang TI, Kam SH, Hu YS, Sharma AA, Lam A, Markowski J, Solimano A, Lavoie PM, Turvey SE (2014). Attenuation of respiratory syncytial virus-induced and RIG-I-dependent type I IFN responses in human neonates and very young children. J Immunol.

[31] Hillyer P, Mane VP, Chen A, Dos Santos MB, Schramm LM, Shepard RE, Luongo C, Le Nouen C, Huang L, Yan L (2017). Respiratory syncytial virus infection induces a subset of types I and III interferons in human dendritic cells. Virology.

[32] Ioannidis I, McNally B, Willette M, Peeples ME, Chaussabel D, Durbin JE, Ramilo O, Mejias A, Flano E (2012). Plasticity and virus specificity of the airway epithelial cell immune response during respiratory virus infection. J Virol.

[33] Miller EK, Hernandez JZ, Wimmenauer V, Shepherd BE, Hijano D, Libster R, Serra ME, Bhat N, Batalle JP, Mohamed Y (2012). A mechanistic role for type III IFN-lambda1 in asthma exacerbations mediated by human rhinoviruses. Am J Respir Crit Care Med.

[34] Giuffrida MJ, Valero N, Mosquera J, de Mon MA, Chacin B, Espina LM, Gotera J, Bermudez J, Mavarez A (2014). Increased cytokine/chemokines in serum from asthmatic and non-asthmatic patients with viral respiratory infection. Influenza Other Respir Viruses.

[35] Rajajendram R, Tham CL, Akhtar MN, Sulaiman MR, Israf DA (2015). Inhibition of epithelial CC-family chemokine synthesis by the synthetic chalcone DMPF-1 via disruption of NF-kappaB nuclear translocation and suppression of experimental asthma in mice. Mediators Inflamm.

[36] Turi KN, Shankar J, Anderson LJ, Rajan D, Gaston K, Gebretsadik T, Das SR, Stone C, Larkin EK, Rosas-Salazar C (2018). Infant viral respiratory infection nasal immune-response patterns and their association with subsequent childhood recurrent wheeze. Am J Respir Crit Care Med.

